# Age-related upregulation of perineuronal nets on inferior collicular cells that project to the cochlear nucleus

**DOI:** 10.3389/fnagi.2023.1271008

**Published:** 2023-11-20

**Authors:** Laila S. Almassri, Andrew P. Ohl, Milena C. Iafrate, Aidan D. Wade, Nick J. Tokar, Amir M. Mafi, Nichole L. Beebe, Jesse W. Young, Jeffrey G. Mellott

**Affiliations:** ^1^Department of Anatomy and Neurobiology, Northeast Ohio Medical University, Rootstown, OH, United States; ^2^Department of Biological Sciences, Kent State University, Kent, OH, United States; ^3^The Ohio State College of Medicine, The Ohio State, Columbus, OH, United States

**Keywords:** inferior colliculus, *Wisteria floribunda* agglutinin, aging, cochlear nucleus (CN), perineuronal nets

## Abstract

**Introduction:**

Disruptions to the balance of excitation and inhibition in the inferior colliculus (IC) occur during aging and underlie various aspects of hearing loss. Specifically, the age-related alteration to GABAergic neurotransmission in the IC likely contributes to the poorer temporal precision characteristic of presbycusis. Perineuronal nets (PNs), a specialized form of the extracellular matrix, maintain excitatory/inhibitory synaptic environments and reduce structural plasticity. We sought to determine whether PNs increasingly surround cell populations in the aged IC that comprise excitatory descending projections to the cochlear nucleus.

**Method:**

We combined Wisteria floribunda agglutinin (WFA) staining for PNs with retrograde tract-tracing in three age groups of Fischer Brown Norway (FBN) rats.

**Results:**

The data demonstrate that the percentage of IC-CN cells with a PN doubles from ~10% at young age to ~20% at old age. This was true in both lemniscal and non-lemniscal IC.

**Discussion:**

Furthermore, the increase of PNs occurred on IC cells that make both ipsilateral and contralateral descending projections to the CN. These results indicate that reduced structural plasticity in the elderly IC-CN pathway, affecting excitatory/inhibitory balance and, potentially, may lead to reduced temporal precision associated with presbycusis.

## 1 Introduction

At the center of all major ascending and descending auditory pathways sits the inferior colliculus (IC). The IC, within its three primary subdivisions (central [ICc], lateral cortex [IClc], dorsal cortex [ICd]), processes information from numerous ascending and descending projections from essentially every station of the central auditory pathway (see review: Thompson, 2005; Wenstrup, 2005). The three IC subdivisions each receive dozens of excitatory and inhibitory inputs, while also send varying combinations of excitation and inhibition to numerous nuclei (e.g., the medial geniculate body [MG], superior colliculus, commissural IC, and superior olivary complex; González-Hernández et al., 1996; Oliver, 2005; Thompson, 2005; Wenstrup, 2005; Mellott et al., 2014a, 2018). The IC also sends a robust descending projection to the cochlear nucleus (CN). However, unlike the aforementioned projections from the IC, the IC-CN pathway is exclusively excitatory and does not include an inhibitory component (Milinkeviciute et al., 2017). Although reports differ across species, broadly speaking, the IC-CN pathway originates from both the lemniscal ICc and non-lemniscal IC (mostly IClc) and targets both dorsal cochlear nuclei (Andersen et al., 1980; Conlee and Kane, 1982; Shore et al., 1991; Caicedo and Herbert, 1993; Malmierca et al., 1996; Saint Marie, 1996; Schofield, 2001; Milinkeviciute et al., 2017). The specific feedback the IC provides to the CN is not completely understood, however this projection was recently demonstrated to target all but one cell type in the DCN (Balmer and Trussell, 2022).

In the IC, as well as nearly every auditory subcortical nucleus, exists populations of perineuronal nets (PNs) that surround non-GABAergic and GABAergic cells (Bertolotto et al., 1996; Hilbig et al., 2007; Foster et al., 2014; Sonntag et al., 2015, 2018; Balmer, 2016; Fader et al., 2016 Beebe et al., 2018; Beebe and Schofield, 2018; Heusinger et al., 2019; Mansour et al., 2019; Sucha et al., 2019; Mafi et al., 2020, 2021). PNs are specialized structures of extracellular matrix molecules that surround the somas and dendrites of various populations of neurons in the central nervous system (Cragg, 1979; Karetko and Skangiel-Kramska, 2009; Sonntag et al., 2015; Bosiacki et al., 2019; Testa et al., 2019). The major molecular components of PNs, chondroitin sulfate proteoglycans (CSPGs), are involved in numerous functions including inhibition of structural plasticity, maintenance of excitatory/inhibitory balance, stabilization of synaptic transmission, and regulation of oxidative stress (Sonntag et al., 2015; Bosiacki et al., 2019; Testa et al., 2019; Mueller-Buehl et al., 2023). PNs are often associated with fast spiking GABAergic interneurons due to their prominence around parvalbumin cells in cerebral cortex (Härtig et al., 1992, 1994; Nowicka et al., 2009). Our understanding of PN function is best understood in cortex with the maturation of GABAergic circuits in the visual system, as they contribute to the timing of the critical period and development of ocular dominance columns (Pizzorusso et al., 2002; Hensch, 2005; Maffei and Turrigiano, 2008; Ye and Miao, 2013). PNs have also been shown to inhibit structural plasticity, and the enzymatic degradation of PNs can promote plasticity in the adult cortex (Pizzorusso et al., 2002, 2006; Gogolla et al., 2009). However, in subcortical nuclei like the IC, PNs also surround excitatory populations (Foster et al., 2014; Beebe et al., 2016; Fader et al., 2016; Mafi et al., 2020, 2021).

During aging, PNs in cerebral cortex are upregulated on parvalbumin-expressing interneurons and populations of non-GABAergic cells (Kosaka and Heizmann, 1989; Härtig et al., 1992; Tanaka and Mizoguchi, 2009; Yamada and Jinno, 2013; Karetko-Sysa et al., 2014; Ueno et al., 2018). Aging is typically associated with increased neuropathology, increased inflammation, and increased microglial activation (Latham et al., 2023). Because microglia can interact with PNs directly and release enzymes responsible for PN degradation, increased microglial activation is associated with decreased PN presence, especially in age-related pathologies like Alzheimer’s (Reinhard et al., 2015; Crapser et al., 2020; Reichelt et al., 2021). As such, age-related changes to cortical populations of PNs has garnered a lot of attention due to the altered plasticity demonstrated in the cerebral cortex across many disease states (e.g., Alzheimer’s Disease, Parkinson’s Disease, autism, epilepsy, traumatic brain injury and schizophrenia; see reviews: Burke and Barnes, 2006; Karetko and Skangiel-Kramska, 2009; Wlodarczyk et al., 2011; Wang and Fawcett, 2012; Soleman et al., 2013; Wen et al., 2018; Bosiacki et al., 2019; Duncan et al., 2019; Liguori et al., 2019; Testa et al., 2019; Ali et al., 2023). Investigations of PNs in auditory cortex have demonstrated changes to PN populations during age that contribute to auditory fear conditioning, fragile X syndrome and auditory plasticity (Brewton et al., 2016; Banerjee et al., 2017; Cisneros-Franco et al., 2018; Ueno et al., 2018; Reinhard et al., 2019; Pirbhoy et al., 2020).

It is well documented that the aging IC undergoes a downregulation of GABAergic neurotransmission (see reviews: Caspary et al., 2008; Syka, 2010, 2020; Caspary and Llano, 2018). GABAergic downregulation in the IC can result from a reduction in GABA synthesis, modification of postsynaptic GABA_A_ receptor subunits, and loss of GABAergic synapses (Syka, 2020). Additionally, the aging IC also experience age-related changes in the distribution of postsynaptic GABA_A_ receptors, downregulate GABAergic synapses, and upregulate PNs (Milbrandt et al., 1996, 1997; Helfert et al., 1999; Robinson et al., 2019; Mafi et al., 2020, 2022). These age-related changes to GABAergic neurotransmission can degrade temporal processing and contribute to presbycusis (Palombi and Caspary, 1996a,b; Walton et al., 1998, 2002; Frisina, 2001; Frisina and Rajan, 2005; Parthasarathy and Bartlett, 2011; Pollak et al., 2011; Cai et al., 2014; Syka, 2020). Despite the numerous synaptic changes occurring in the aging IC, we have little knowledge regarding which of the many IC circuits, in particular the descending circuits, undergo such changes.

In the current study, based on our previous work, we hypothesize that the IC-CN pathway is increasingly surrounded by PNs with age (Mafi et al., 2020). We combined retrograde tract-tracing with *Wisteria floribunda* agglutinin (WFA) staining to identify IC-CN neurons that were surrounded by PNs in three age groups of Fischer Brown Norway (FBN) rats. To maintain consistency with our previous studies on the aging IC and the many studies on changes to inhibitory neurotransmission in the aging auditory system we use The National Institute on Aging’s FBN rat as an aging model due to its long median life span (Milbrandt and Caspary, 1995; Ling et al., 2005; Turner et al., 2005; Caspary et al., 2008; Wang et al., 2009; Hughes et al., 2010; Richardson et al., 2011, 2013; Cai et al., 2018). We sought to determine whether (1) IC-CN cells were surrounded by PNs and (2) whether the presence of PNs changed in the IC-CN pathway over the adult lifespan. We use three age groups: a young group at 3–5 months of age; a middle-age group at 18–20 months of age; and an old group at 26–28 months of age, which is when age-related hearing deficits are routinely present in the FBN rat (Cai et al., 2018). We believe that this is the first study addressing PNs on an excitatory descending subcortical circuit. We found that (1) a population of ICc-CN and IClc-CN cells at young age had a PN and projections of these cells targeted the ipsilateral and contralateral CN, and (2) the percentage of IC-CN cells with a PN significantly increases at old age.

## 2 Materials and methods

### 2.1 Animals

All procedures were conducted in accordance with the Northeast Ohio Medical University Institutional Animal Care and Use Committee and NIH guidelines. Results are described from 14 male FBN rats (National Institute of Aging; Bethesda, MD, United States; RRID:SCR_007317) across three age groups: 3–5 months “young” weighing 286-402 g; 18–20 months “middle-age” weighing 499-580 g; 26–28 months “old” weighing 572-604 g. Efforts were made to minimize the number of animals and their suffering.

### 2.2 Surgery and retrograde tracers

Each animal was anesthetized with isoflurane (4–5% for induction, 1.75–2% for maintenance; Butler Schein, Dublin, OH, United States) in oxygen and the head positioned in a stereotaxic frame. An incision was made in the scalp and the surrounding skin was injected with a long-lasting local anesthetic (0.25% bupivacaine with epinephrine 1:200,000; Hospira, Inc., Lake Forest, IL, United States). A surgical drill was used to make a craniotomy over the desired interaural target coordinates (left CN; AP = −2.2/−2.3, ML = 3.5/3.6, DV = 2.6/2.7: right CN; AP = −2.2/−2.3, ML = −3.5/−3.6, DV = 2.6/2.7). Following the tracer injection, Gelfoam (Harvard Apparatus, Holliston, MA, United States) was placed in the craniotomy site and the scalp was sutured. Meloxicam ER (meloxicam, 4 mg/kg s.c.; ZooPharm, Laramie, WY, United States) was administered to provide post-operative analgesia. The animal was monitored until it could walk, eat and drink without difficulty.

Fluorescent tracers FluoroGold (4% in water), FluoroChrome, Inc., Englewood, CO, United States; red fluorescent RetroBeads (not diluted) [“red beads”], Luma-Fluor, Inc., Naples, FL, United States; green fluorescent RetroBeads (not diluted) [“green beads”], Luma-Fluor, Inc., Naples, FL, United States; FluoroRuby tetramethylrhodamine dextran, 3000 molecular weight, (Invitrogen, Eugene, OR, United States) were deposited in the CN with a Hamilton microsyringe (1 μL; Hamilton, Reno, NV, United States) positioned according to stereotaxic coordinates (Table 1). Each tracer was injected with a different syringe at 1–2 sites (depending on the diffusibility of the tracer, Schofield, 2008) in order to include as much of the CN as possible while limiting the spread of tracer into neighboring nuclei. We attempted to inject a total of twenty-six CNs (13 cases × 2 CNs). Eight cases had one rejected injection due to missing the CN or the tracer spread too far.

**Table 1:** Summary of cases, ages, tracer deposits and the number of IC-CN cells that were retrogradely labeled and the percentage of cells with a PN across the ICc and IClc.

Case	Age (mo)	Sex	Left CN tracer deposits total volume	Right CN Tracer Deposits Volume	# of ICc-CN cells	% of ICc-CN cells w/PN	# of Ipsi ICc-CN cells w/PN	% of Ipsi ICc-CN cells w/PN	# of Contra ICc-CN cells w/PN	% of Contra ICc-CN cells w/PN	# of IClc-CN cells	# of IClc-CN cells w/PN	% of IClc-CN cells w/PN	# of Ipsi IClc-CN cells w/PN	% of Ipsi IClc-CN cells w/PN	# of Contra IClc-CN cells w/PN	% of Contra IClc-CN cells w/PN
R72	3	M	FG^#^, 1, 0.1 μL	FR, 1, 0.2 μL	278	10.1	152	10.5	126	9.5	83	6	7.2	38	7.9	45	6.7
R74	3	M	FG^#^, 1, 0.1 μL	FR, 1, 0.2 μL	579	11.2	383	10.2	196	13.2	251	30	12	159	10.7	92	12.1
R91	3	M	GB, 1, 0.2 μL	RB, 1, 0.2 μL	132	6.8	67	5.9	65	8	44	2	4.5	25	8	19	5.3
R56	5	M	FG^#^, 1, 0.1 μL	FR, 1, 0.2 μL	663	8.7	368	9	295	5.1	377	28	7.4	194	7.2	183	6
Totals					1,652	9.7	970	10.4	682	8.5	755	66	8.7	416	9.1	339	8
R151	18	M	FG^#^, 1, 0.1 μL	FR, 1, 0.2 μL	101	19.8	53	9.4	48	18.8	34	4	11.8	23	17.4	11	0
R155	18	M	FG^#^, 1, 0.1 μL	FR, 1, 0.2 μL	149	17.4	82	17.1	67	12.9	55	6	10.9	35	11.4	20	10
R160	18	M	FG^#^, 1, 0.1 μL	FR, 1, 0.2 μL	233	12	112	14.3	121	9.9	83	7	8.4	55	3.6	28	7.1
R161	18	M	FG^#^, 1, 0.1 μL	FR, 1, 0.2 μL	338	10.7	173	10.9	164	11	86	7	8.1	66	7.6	21	9.5
R65	20	M	FG^#^, 1, 0.1 μL	FR, 1, 0.2 μL	248	12.5	126	7.9	123	17.1	102	8	7.8	24	16.7	77	5.2
Totals					1,069	13.2	546	12.8	523	13.4	360	32	8.9	203	9.4	157	8.3
R54	26	M	FG^#^, 1, 0.1 μL	FR, 1, 0.2 μL	894	17.8	505	21	388	11.6	327	47	14.4	208	13.5	119	14.3
R57	27	M	FG^#^, 1, 0.1 μL	FR, 1, 0.2 μL	350	21.1	186	22.6	164	19.5	75	16	21.3	27	22.2	48	14.6
R77	27	M	FG^#^, 1, 0.05 μL	RB, 1, 0.2 μL	307	22.8	220	20.9	87	25.3	103	22	21.4	73	19.2	30	26.7
R78	28	M	FG^#^, 1, 0.05 μL	RB, 1, 0.2 μL	93	23.7	45	22.2	49	20.4	21	5	23.8	16	18.6	5	20
Totals					1,644	19.8^*^	956	21.3	688	17.2	526	90	17.1^**^	324	15.7	202	18.8

^*^Indicates *p* < 0.05 as compared to 3–5 months “young”. ^**^Indicates *p* < 0.0.01 as compared to 3–5 months “young”. ^#^Indicates FluoroGold diluted in 4% water.

### 2.3 Perfusion and tissue processing

Five to seven days after surgery, each animal was deeply anesthetized with isoflurane and perfused transcardially with 0.9% saline solution, followed by 250 mL of 4% paraformaldehyde in 0.1 M phosphate buffer, pH 7.4 and then by 250 mL of the same fixative with 10% sucrose. The brain was removed and stored overnight at 4°C in fixative with 25–30% sucrose for cryoprotection. The next day the brain was prepared for processing by removing the cerebellum and cortex and blocking the remaining piece with transverse cuts posterior to the cochlear nucleus and anterior to the thalamus (Mafi et al., 2020). The tissue was frozen and cut on a sliding microtome or cryostat into 40 μm thick transverse sections that were collected in six series.

It is common practice for our lab to employ immunochemistry for glutamic acid decarboxylase (GAD) to label GABAergic IC cells. As such, we did for five (two young, 1 middle, 2 old) of the cases in the current study to examine at least one case from each age group. However, as the IC-CN projection is excitatory, none of the 900+ IC-CN cells in these five cases were GAD-positive. Thus, we ceased processing for GAD in the remaining cases. Our process and protocol for GAD immunohistochemistry and quantification can be found in a number of our previous reports (Mellott et al., 2014a,b,c). To label PNs the tissue was pretreated with 0.2% Triton X-100 for permeabilization, washed in phosphate-buffered saline (PBS), and then stained with biotin-tagged *Wisteria floribunda* Lectin (WFA; 1:100; Vector Laboratories; Cat# B1355) and tagged with a streptavidin AlexaFluor in near infrared (Molecular Probes, AF647 [Cat# S-21374] or AF750 [Cat# S21384]). We use near infrared markers (AF647 & AF750) when possible to help minimize autofluorescent signals from lipofuscin (a pigment that accumulates in the cytoplasm during aging) and is prominent in non-near infrared channels (Robinson et al., 2019). After PNs were stained, each section was washed in PBS and incubated for 20 min in a green (500/525) NeuroTrace (NT; #N-21480 Molecular Probes diluted to 1:100) to help the reconstruction of IC subdivisions along with a rat anatomical atlas of the brain (Paxinos and Watson, 1998). Sections were mounted on gelatin-coated slides, allowed to dry and coverslipped with DPX (Sigma).

### 2.4 Data analysis

Sections were chosen through the mid-rostrocaudal region (between interaural levels −0.12–0.60 mm; Paxinos and Watson, 1998) of the IC in which the three major IC subdivisions (central IC [ICc], lateral cortex of the IC [IClc] and dorsal cortex of the IC [ICd]) are present. This resulted in four IC sections per case. Consistent with previous studies of IC-CN projections, we found retrogradely labeled cells were primarily distributed across the ipsilateral and contralateral central IC (ICc) and the ipsilateral and contralateral lateral cortex of the IC (IClc; Schofield, 2001; Thompson, 2005; Milinkeviciute et al., 2017). Similar to Milinkeviciute et al. (2017) we also found retrogradely labeled cells in the dorsal IC (ICd), but at lesser numbers. However, ICd-CN cells rarely had a PN and were left out of the data analysis from the current study. We initially subdivided (with Neurolucida) the ventromedial-dorsolateral axis of the ICc into three equal lengths to create anatomical regions representing the distribution of high, middle and low frequencies. However, analysis showed no significance across these three regions regarding the likelihood that a ICc-CN cell was netted at any age. Each IC-CN cell was identified as either having a PN or not having a PN (Foster et al., 2014; Beebe and Schofield, 2018; Mafi et al., 2020, 2021). Briefly, we used a Neurolucida reconstruction system (MBF Bioscience, Williston, VT, United States) to determine the extent to which the perimeter of each IC-CN cell was encircled by aggregated extracellular matrix. If a cell was partially encircled by the extracellular matrix, we used a threshold of 90% perimeter coverage to define a PN (Beebe et al., 2016; Mafi et al., 2020).

Tissue samples stained for WFA from thirteen animals were chosen for quantification. Even though the number of animals in the current study was lower than our previous studies of PNs in the aging IC, the data was extremely consistent from case to case and significance was reached in our old animals. Four transverse IC sections (ipsilateral to the injected CN), in a series of tissue containing the three major IC subdivisions (ICc, IClc and ICd), was quantified per experiment. Thus, each section plotted was spaced 240 μm (6 series × 40 μm thick sections) from the neighboring sections that were quantified, guaranteeing that no cell was double-counted. Every retrogradely-labeled cell in the ICc and the IClc was quantified. Each retrogradely-labeled IC-CN cell that did or did not have a PN was manually plotted with a 63x oil-immersion objective (numerical aperture 1.4) aligned to a Neurolucida reconstruction system (MBF Bioscience, Williston, VT, United States) attached to a Zeiss AxioImager M2. Each combination was plotted with a unique marker. Plots of markers and IC subdivisions were then analyzed through NeuroExplorer (MBF Bioscience, Williston, VT, United States). All photomicrographs presented here are 2–5 μm maximum image projections and were captured with a Zeiss AxioImager M2 fluorescence microscope and Hamamatsu Orca Flash 4.0 camera (Hamamatsu) and optically sectioned at 0.5 μm steps with an Apotome 2 (Zeiss). Adobe Photoshop (Adobe Systems) was used to add scale bars, crop images, erase background around tissue sections, adjust intensity levels and colorize monochrome images. The results of these plots were used for a quantitative summary of the distribution of PNs surrounding IC-CN cells. Final images of the plots were refined with Adobe Illustrator (Adobe Systems, Inc., San Jose, CA, United States) for preparation of figures.

Variation in the occurrence of IC-CN cells surrounded by a PN according to age group and IC subdivision was analyzed using linear mixed-effects models. Mixed-effects models allow for a hybrid of repeated measures analysis (i.e., “within-subject” variables), Model I ANOVA fixed factor analysis (i.e., “between-subject” variables), and Model II ANOVA random factor analysis (i.e., variance components), in the same gestalt statistical test. In this study, age group was specified as a between-subjects fixed factor across individual rats, individual IC subdivision and IC-CN cells were specified as within-subjects fixed factors within individual rats, and individual rat number was specified as a random factor. Outliers to fitted models were examined by calculating Cook’s distances, identifying outliers as those pints with Cook’s distances greater than 4/n, where n is model sample size. Though 2–3 data points in each model qualified as outliers, by this definition, refitting models without these points had little influence on statistics and resulting *p*-values, requiring no change in model interpretation *p*-values for pairwise *post hoc* tests of differences between consecutive age groups (i.e., 3–5 months versus 18–20 months, 18–20 months versus 26–28 months) were adjusted using the False Discovery Rate procedure (Benjamini and Hochberg, 1995), a method that simultaneously limits experiment-wise alpha inflation and minimizes the correlated loss of statistical power. All statistical tests were performed in R (version 3.6.3 for Mac OS X; R Core Team, 2020), supplemented by the add-on packages *nlme* (Pinheiro et al., 2019) and *emmeans* (Lenth, 2019).

## 3 Results

**Figure 1:**
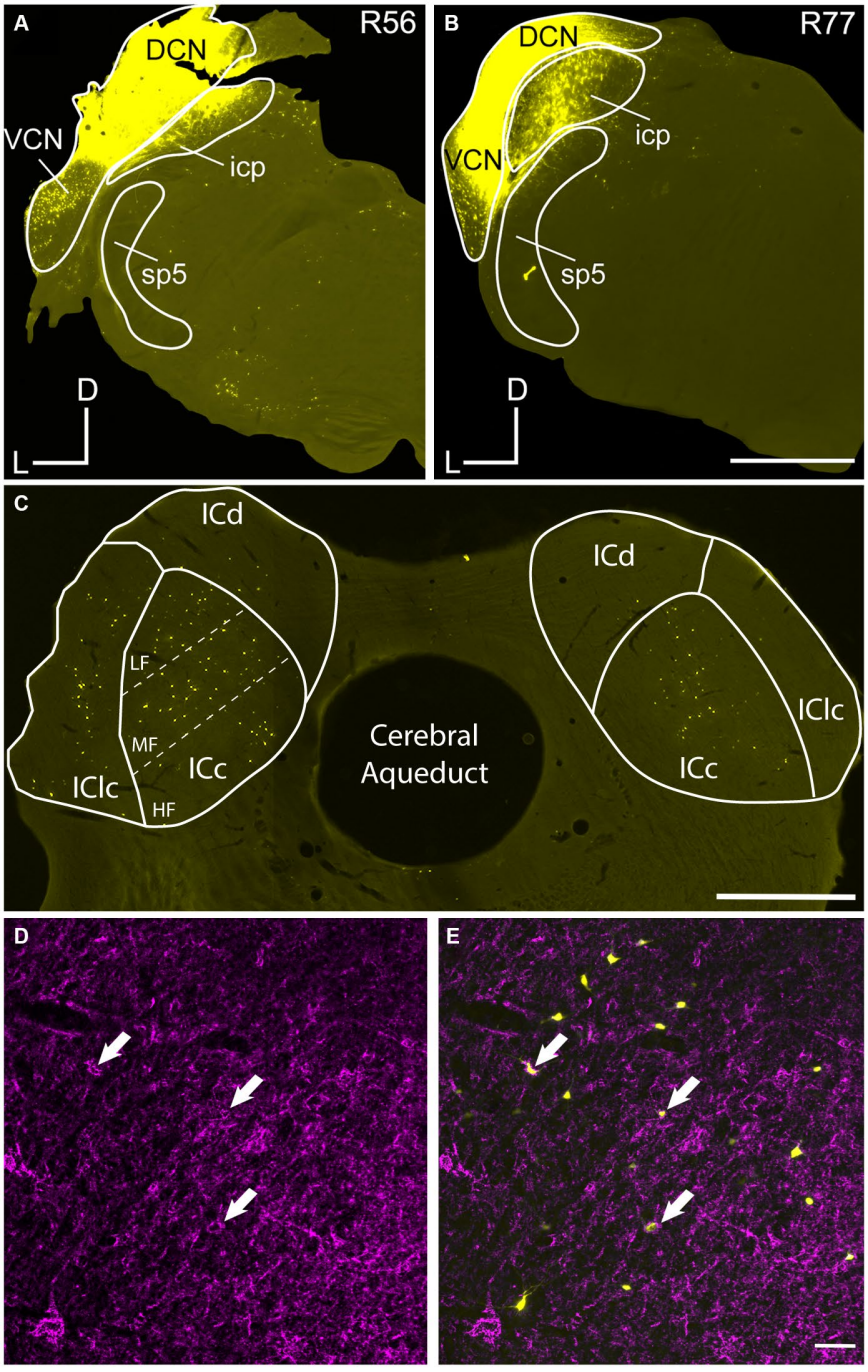
Injection sites of retrograde tracers deposited in the cochlear nucleus (CN). Photomicrographs show representative young and old cases with large deposits of FluoroGold (FG) in the CN. **(A)** Deposits of FG in the left CN of a 5 month old FBN rat (R56). **(B)** Deposit of FG in the left CN of a 27 month old FBN rat (R77). **(C)** Low mag view of retrogradely labeled cells across the ipsilateral (left) and contralateral (right) IC as a result of the deposit made in **(A)**. Dashed lines represent the dorsolateral to ventromedial axis subdivided into low frequency (LF), middle frequency (MF) and high frequency (HF) regions across the ICc. **(D,E)** PNs in the ipsilateral ICc from **(B)**. Arrows point to three PNs that surround cells labeled with FG. DCN, dorsal cochlear nucleus; ICc, central nucleus of the IC; ICd, dorsal cortex of the IC; IClc, lateral cortex of the IC; icp, inferior cerebellar peduncle; sp5, spinal trigeminal tract; VCN, ventral cochlear nucleus; D, dorsal; L, lateral; M, medial. Scale bars: **A–C** = 1 mm; **D,E** = 50 μm.

Figure 1 shows tracer deposits in the CN in a representative experiment in each age group and accompanying images demonstrating the distribution of retrogradely labeled cells, and retrogradely labeled cells with PNs. Tracer deposits in each animal were made using the same window of stereotaxic coordinates (listed in Methods). The retrograde tracers used and the volume deposited was kept largely consistent across each animal in the current study (Table 1). Each injection labeled a large number of cells in the ipsilateral and contralateral IC. We found retrogradely labeled cells were primarily distributed across the central IC (ICc) and the lateral cortex of the IC (IClc; Figure 1). Lesser numbers of retrogradely labeled cells were present in the dorsal IC (ICd) but rarely had a PN at any age. As such we do not include analysis of the ICd in the current results. Unlike our previous study on netted IC cells that project to the auditory thalamus, we did not find a robust decrease in the number of overall cells retrogradely labeled in the old age groups (Table 1; Mafi et al., 2021).

We combined retrograde tracing to identify IC-CN cells with WFA staining for PNs. Deposits of retrograde tracer were designed to be “large” as the tracer was deposited into the major subdivisions of the CN in each case (Figure 1). Occasionally our tracer deposits would clip the inferior cerebellar peduncle (icp), which is located medially to the CN (Figure 1). However, we do not believe that deposits including the icp confound our data as axons of IC cells are not found in the icp.

We present the percentage of IC-CN cells that have a PN in the ICc and IClc at each age. Results are described from 6,006 retrogradely-labeled cells (4,365 ICc-CN cells; 1,641 IClc-CN cells) across 13 cases, 4–5 per age group (Table 1). Overall, the IC to the CN projection had a slight ipsilateral dominance as 3,415 of the 6,006 retrogradely labeled cells (56.9%) were located in the ipsilateral IC. Of the 4,365 ICc-CN cells, 2,472 (56.7%) of them were ipsilateral. Of the 1,641 IClc-CN cells, 943 (57.5%) were ipsilateral. While deposits of the same retrograde tracer at the same volume may not label the same number of cells between experiments, the total number of cells labeled in the young and old ICc was remarkably similar (young: 1,652 vs. old: 1,644; Table 1). We interpret these similarities too indicate that age may not affect the mechanisms of retrograde transport in the IC-CN pathway.

### 3.1 Percentage of IC-CN cells surrounded by a PN increases with age

Retrogradely labeled cells with a PN were readily identified in the IC of each age group. We found that with aging the percentage of IC-CN cells surrounded by a PN significantly increased from 9.4% in the young group to 19.1% in the old group (Table 1). In contrast to our previous study of PNs surrounding IC cells that project to the auditory thalamus, although there was an increasing trend, there was no significant increase found during middle age in either subdivision (Table 1; Mafi et al., 2021). We also examined the IC-CN population across the ipsilateral and contralateral IC (Table 1). Given that the percentages of ipsilateral and contralateral IC-CN cells with a PN were remarkably similar to the overall population (young IC-CN – 9.4%; young ipsilateral IC-CN – 9.3%; young contralateral IC-CN – 8.3%; old IC-CN – 19.1%; old ipsilateral IC-CN 18.8%; old contralateral IC-CN – 17.8%), we chose to treat the ipsilateral and contralateral populations as one to increase brevity and conciseness. We detail trends in the ICc and the IClc below.

#### 3.1.1 Central nucleus of the IC

**Figure 2:**
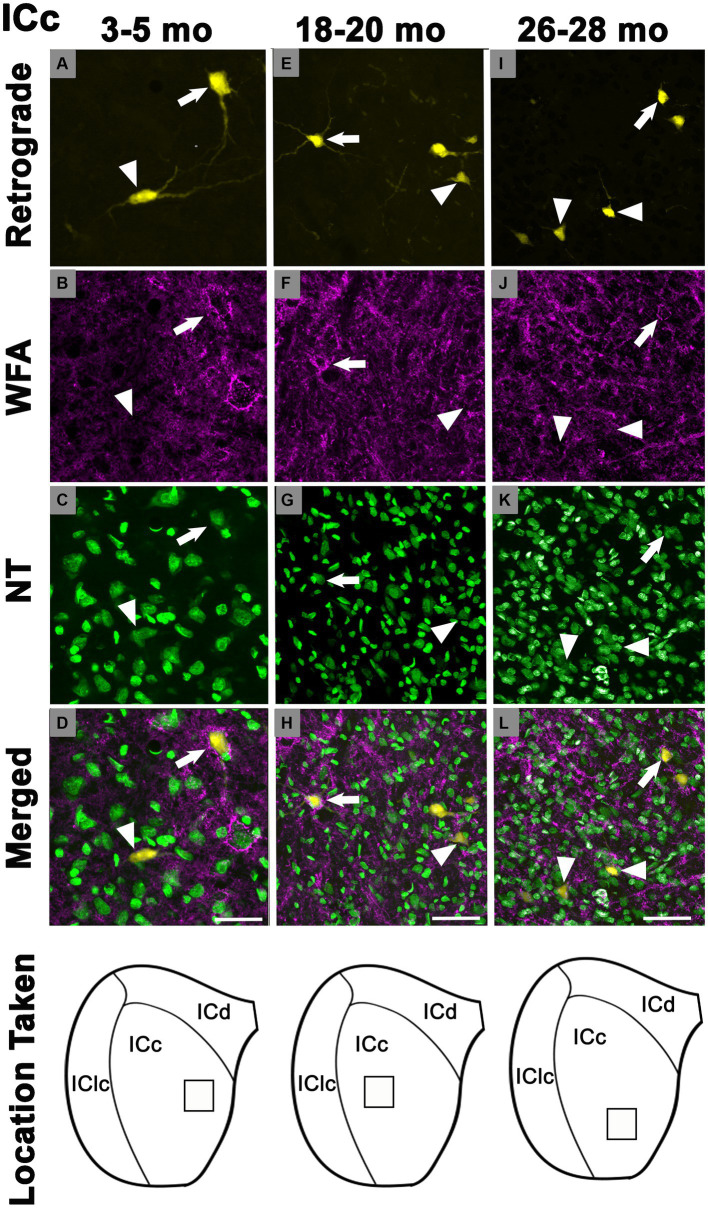
Structured illumination fluorescence images, taken at 0.5 μm steps, showing cells in the central inferior colliculus that project to the cochlear nucleus with (arrows) or without (arrowheads) a PN across three ages. In the first row, cells retrogradely-labeled with FluoroGold shown in yellow. PNs are shown in magenta or cyan in the second row. NeuroTrace is shown in green in the third row. The fourth row shows the merged image of the retrograde, WFA, and NeuroTrace. Each column represents the ICc across a single age group. **(A–D)** Photomicrographs (presented at a higher magnification than E-L to better demonstrate the dendritic filling with FluoroGold) of a 3 month old ICc. Case R74. **(E–H)** Photomicrographs of an 18 month old ICc. Case R161. **(I–L)** Photomicrographs of 27 month old ICc. Case R77. The last row shows a schematic with a square demonstrating where in the ICc the photomicrographs were taken. Scale bars: D = 20 μm; H&L = 50 μm.

**Figure 3:**
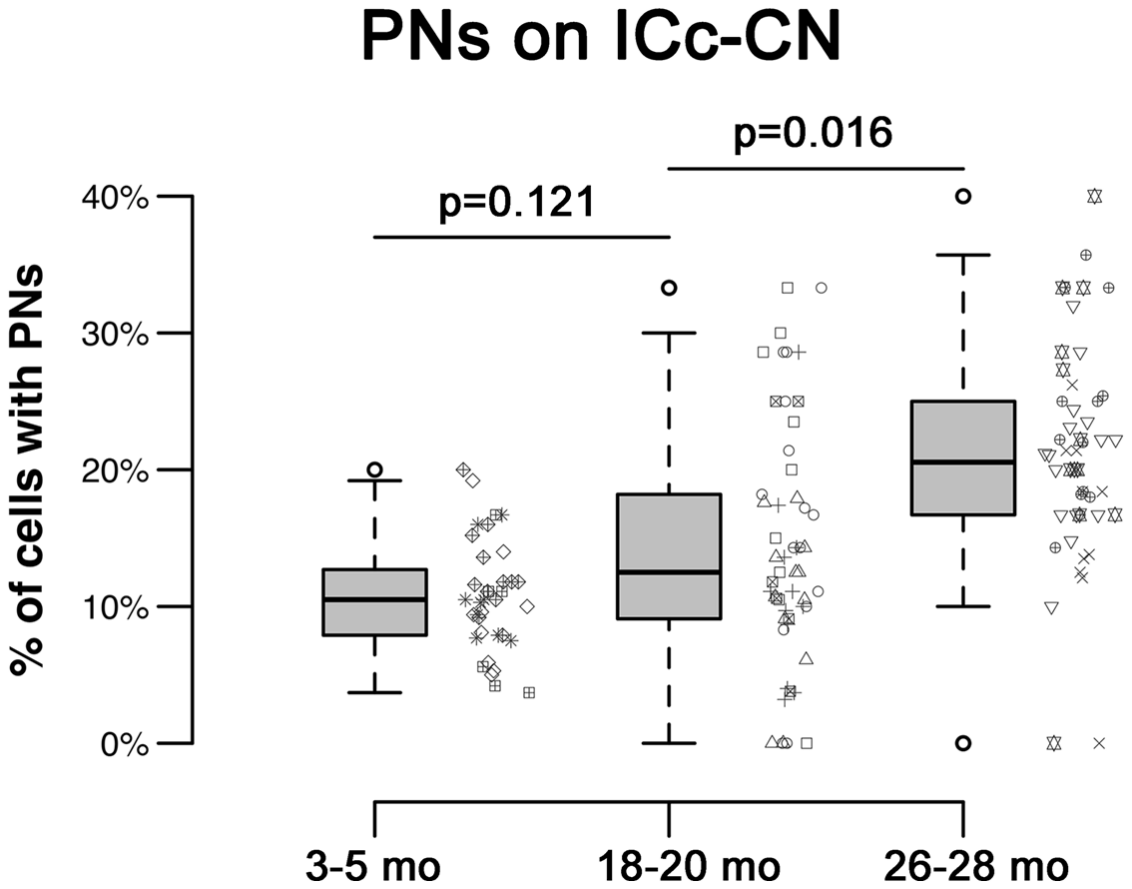
Box plots summarizing the percentage of ICc-CN cells surrounded by a PN across three age groups. Pairwise differences demonstrated a significant, increase between the 18–20 month and 26–28 month age groups (*p = 0.016). Pairwise differences demonstrated no significant differences between the 3–5 month and 18–20 month age groups (*p* = 0.121). Unique symbols to the right of each box plot represent the individual cases. In each box plot, dark lines represent the median of the distribution, boxes extend across the interquartile range, and whiskers extend to ±150% of the interquartile range. Circles indicate outliers beyond this range.

In each age group, PNs surrounded a subpopulation of retrogradely-labeled ICc-CN cells (Figure 2; with a PN, arrows; without a PN, arrowheads). PN-surrounded ICc-CN cells varied in size, however most netted ICc-CN cells had a diameter less than 20 μm (Figure 2; compare arrows in C, F, & I). Across the young cases the percentages of ICc-CN cells that had a PN ranged from 6.8 to 11.2% (Table 1). During middle age, the percentages of ICc-CN cells that had a PN ranged from 10.7 to 19.8% (Table 1). In old age the percentages ranged from 17.8 to 23.7% (Table 1). The percentage of ICc-CN cells that were netted did not significantly increase from 3–5 months (9.7%) to 18–20 months (13.2%, *p* = 0.121; Figure 3). The percentage of ICc-CN cells that were netted did significantly increase from 18–20 months (13.2%) to 26–28 months (19.8%, *p = 0.016; Figure 3). As stated in the Methods, we initially analyzed retrogradely labeled cells with and without a PN based on their location in the high, middle, and low anatomically defined frequency regions of the IC. However, we found that the location of a given netted cell across the ventromedial-dorsolateral ICc axis was not a factor in the age-related increase of PNs on ICc-CN cells (*p* = 0.679). We conclude that PNs within the ICc-CN descending pathway (1) significantly increase during old age and (2) and are uniformly upregulated across the ICc.

#### 3.1.2 Lateral cortex of the IC

**Figure 4:**
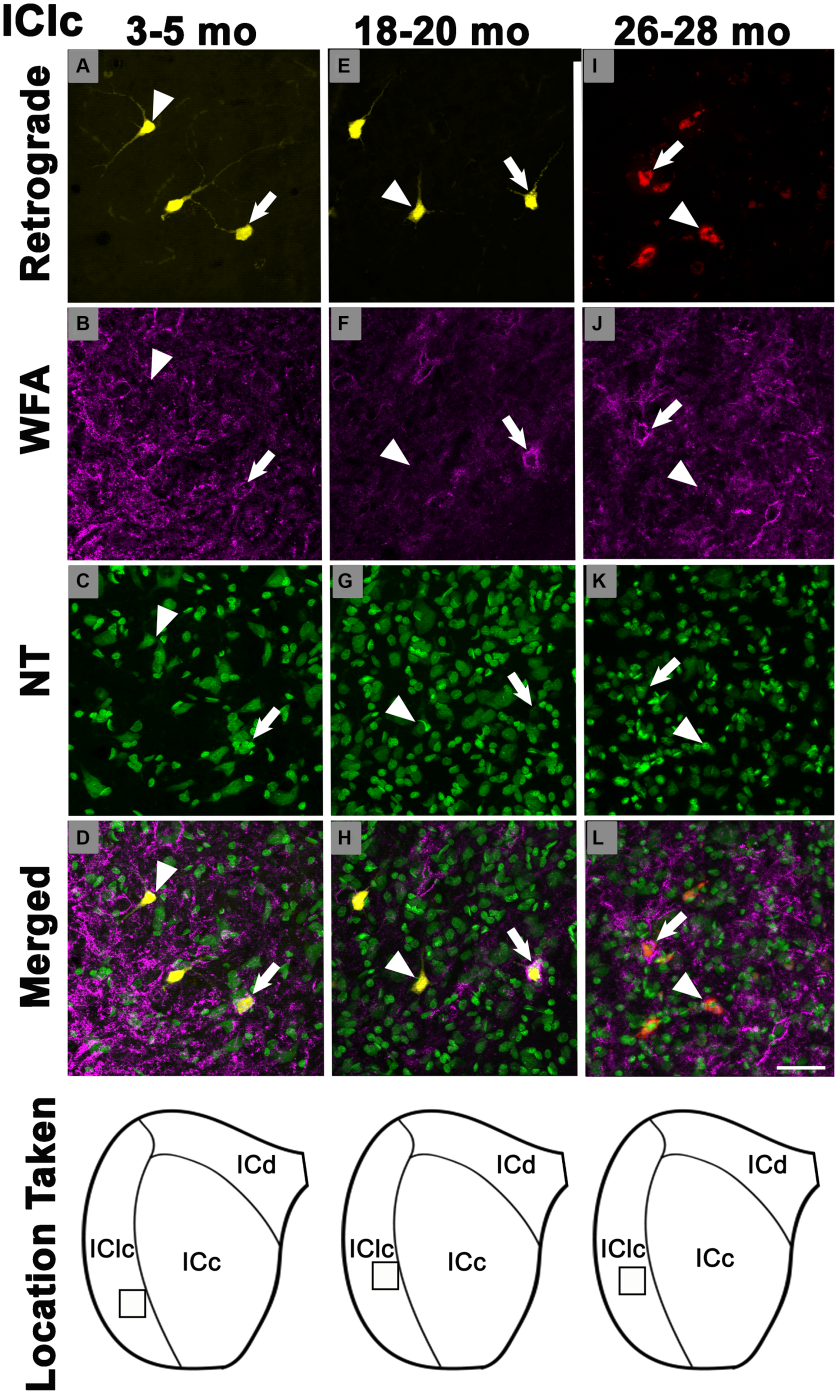
Structured illumination fluorescence images, taken at 0.5 μm steps, showing cells in the lateral cortex of the inferior colliculus that project to the cochlear nucleus with (arrows) or without (arrowheads) a PN across three ages. In the first row, cells retrogradely-labeled with FluoroGold or FluoroRuby are shown in yellow and red, respectively. PNs are shown in magenta in the second row. NeuroTrace is shown in green in the third row. The fourth row shows the merged image of the retrograde, WFA, and NeuroTrace. Each column represents the ICc across a single age group. **(A–D)** Photomicrographs of a 3 month old ICc. Case R72. **(E–H)** Photomicrographs of an 18 month old ICc. Case R160. **(I–L)** Photomicrographs of 27 month old ICc. Case R57. The last row shows a schematic with a square demonstrating where in the IClc the photomicrographs were taken. Scale bar = 50 μm.

**Figure 5:**
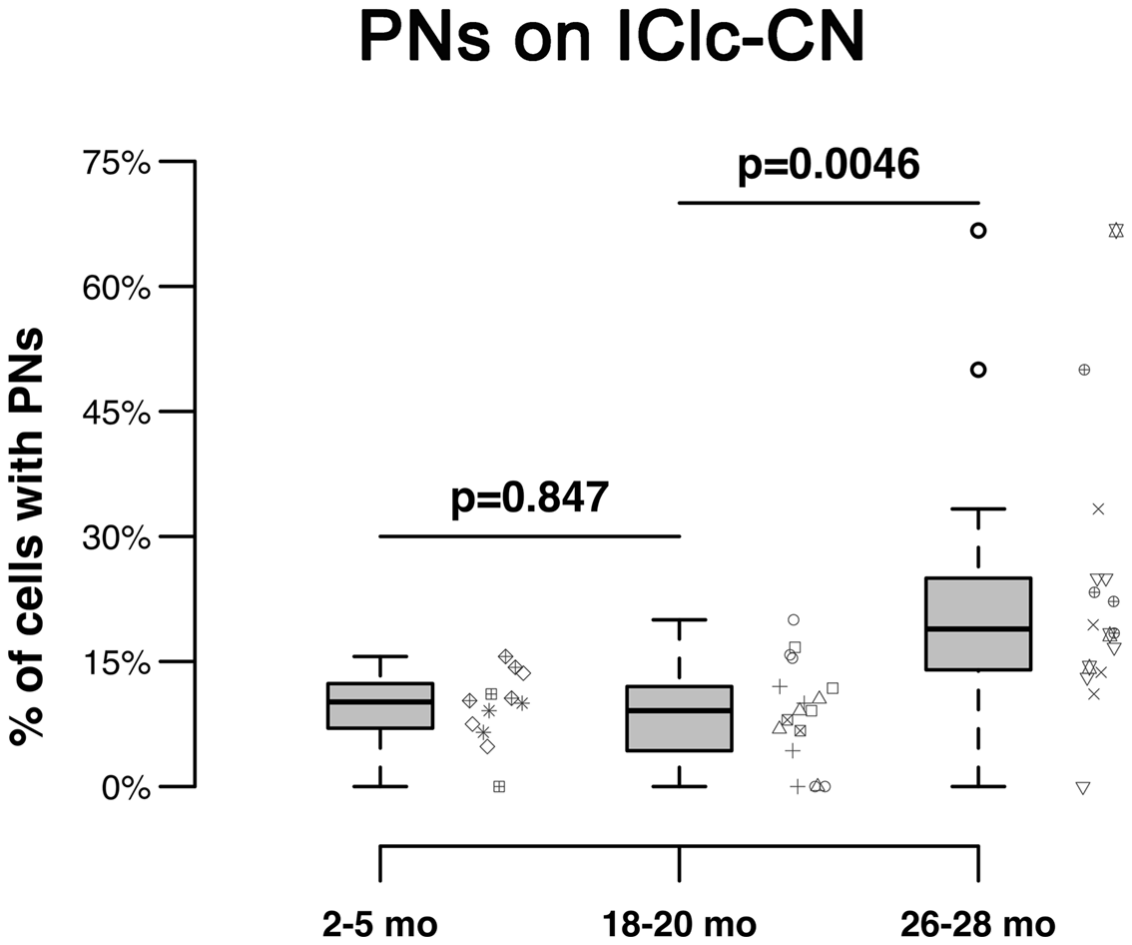
Box plots summarizing the percentage of IClc-CN cells surrounded by a PN across three age groups. Pairwise differences demonstrated a significant, increase between the 18–20 month and 26–28 month age groups (*p = 0.0046). Pairwise differences demonstrated no significant differences between the 3–5 month and 18–20 month age groups (*p* = 0.847). Unique symbols to the right of each box plot represent the individual cases. In each box plot, dark lines represent the median of the distribution, boxes extend across the interquartile range, and whiskers extend to ±150% of the interquartile range. Circles indicate outliers beyond this range.

PNs also surrounded a subpopulation of retrogradely-labeled IClc-CN cells (Figure 4). Similar to netted ICc-CN cells, netted IClc-CN cells varied in size, typically had a diameter less than 20 μm (Figure 4). Across the young group the percentages of IClc-CN cells that had a PN ranged from 4.5 to 12% (Table 1). During middle age, the percentages of IClc-CN cells that had a PN ranged from 7.8 to 11.8% (Table 1). In old age the percentages ranged from 14.3 to 23.8% (Table 1). The percentage of IClc-CN cells that were netted did not significantly increase from 3–5 months (8.7%) to 18–20 months (8.9%, *p* = 0.847; Figure 5). The percentage of IClc-CN cells that were netted did significantly increase from 18–20 months (8.9%) to 26–28 months (17.1%, *p = 0.0046; Figure 5). We conclude that PNs significantly increase in the IClc-CN descending pathway in old age.

### 3.2 Distribution of netted IC-CN cells

**Figure 6:**
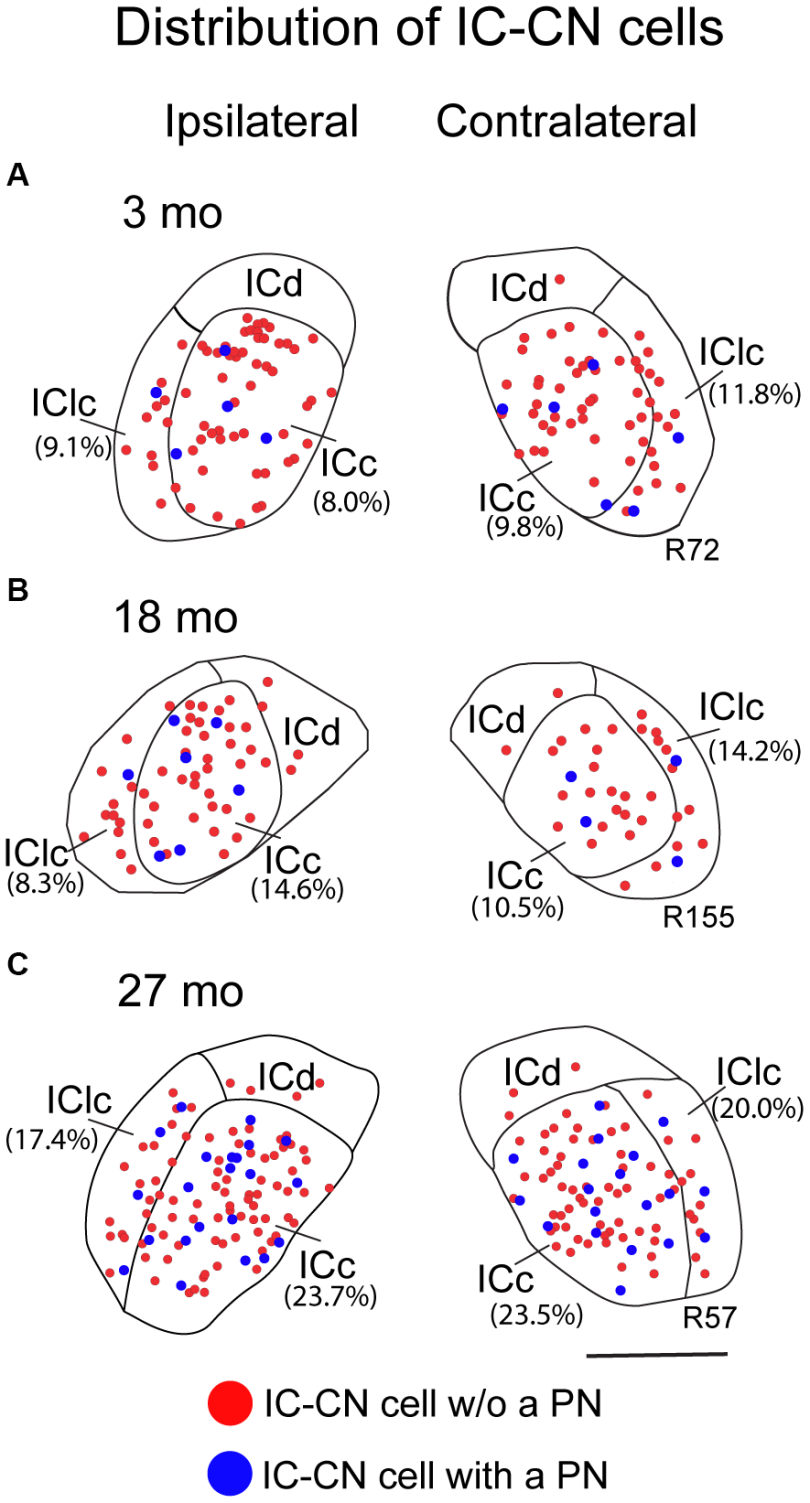
Plots showing the distribution of ipsilateral and contralateral IC-CN cells that have a PN (blue circles) and do not have a PN (red circle). **(A)** Plots of IC-CN cells with and without a PN resultant from a FluoroRuby deposit at 3 months of age; R72. **(B)** Plots of IC-CN cells with and without a PN resultant from a FluoroGold deposit at 18 months of age; R155. **(C)** Plots of IC-CN cells with and without a PN resultant from a FluoroGold deposit at 27 months of age; R57. Each symbol represents one IC-CN cell. Percentage of IC-CN cells with a PN for the individual sections are shown in parentheses. Plotted sections were chosen between interaural levels 0.12–0.48 mm; Paxinos and Watson, 1998. Transverse sections at a mid-rostrocaudal level of the IC. Scale bar = 1 mm.

Figure 6 shows the distribution of IC-CN cells with and without a PN. Netted (blue dots) and non-netted (red dots) IC-CN cells were broadly distributed and intermingled across the ICc and the IClc at each age (Figure 6). This pattern of distribution was consistent between the ipsilateral and contralateral IC at each age (Figure 6). Figure 6 also demonstrates that the percentage of IC-CN cells that had a PN was very similar between the ipsilateral and contralateral IC at each age (Figure 6; compare percentages in parentheses). As mentioned above, retrogradely labeled cells were uncommon if not absent in the ICd. When ICd labeled cells were present, they were not netted (Figure 6).

## 4 Discussion

The current study qualitatively and quantitatively assesses the occurrence of PNs on IC cells that project to the CN during young, middle and old age. The primary finding is that the number of IC-CN cells that have a PN roughly doubles from young/middle age (~9%) to old age (~18%). It appears that the increase of PNs on IC-CN cells was subdivision independent, as the percentage of PNs surrounding ICc-CN and IClc-CN at each age was similar (Table 1). As in our previous reports on PNs in the aging IC we use an age group (18–20 months) to represent a middle age when characteristics of age-related hearing loss are not present in the FBN rat (Cai et al., 2018; Mafi et al., 2020, 2021). We did not find a significant increase in PNs in the IC-CN pathway at this middle age. Thus, changes to structural and synaptic plasticity of IC-CN cells likely occur during and/or after the onset of presbycusis. Before further discussion of our results as they pertain to previous studies and auditory function during aging, we consider a few technical issues regarding our model, staining PNs and PN distribution.

### 4.1 Technical considerations

We use WFA to label PNs as it is an accepted marker for the majority of PNs in the brain, commonly used in the auditory system, and has been used in numerous species (Belichenko et al., 1999; Härtig et al., 1999; Cant and Benson, 2006; Ajmo et al., 2008; Morawski et al., 2012; Foster et al., 2014; Sonntag et al., 2015; Brewton et al., 2016; Beebe and Schofield, 2018; Mafi et al., 2020, 2021). However, as the molecular composition of a given PN in a given nucleus may vary, antibodies to CSPG molecules (aggrecan, brevican, phosphacan) may label populations of PNs that WFA does not reveal (Lurie et al., 1997; Friauf, 2000; Matthews et al., 2002; Brückner et al., 2003; Ajmo et al., 2008; Giamanco et al., 2010; Karetko-Sysa et al., 2011, 2014; Wang and Fawcett, 2012; Blosa et al., 2013; Sonntag et al., 2015). Future studies will hopefully determine the CSPG makeup of PNs that are increased with age in the IC-CN pathway.

Previous studies have found that projections from the IC to the CN are excitatory (Milinkeviciute et al., 2017; Balmer and Trussell, 2022). However, a report in rat indicated that inhibitory IC cells contact DCN cells (Alibardi, 2002). To investigate the neurotransmitter makeup of the IC-CN pathway in our model we processed a subset of cases for GAD immunohistochemistry as we have previously (Mafi et al., 2020, 2021). In the first four cases (2 young, 1 middle, and 1 old) we found no convincing evidence that any of our IC-CN cells were GAD-positive and ceased processing the remaining cases for GAD.

The age groups in the current study were chosen to mirror the age groups from our previous studies on the aging IC in the FBN rat (Mafi et al., 2020, 2021, 2022; Koehler et al., 2023). The middle age group of 18–20 months reflects a period of time when significant pre- and postsynaptic changes occur without significant age-related changes to hearing thresholds in the FBN (Helfert et al., 1999; Caspary et al., 2008; Cai et al., 2018; Robinson et al., 2019). However, unlike our previous studies (discussed further below), we did not find significant changes within the IC during middle age and, as such, we interpret the increase of PNs in the IC-CN pathway as occurring during and/or after the onset of severe presbycusis in the FBN rat.

### 4.2 Comparison to previous studies

Given that PNs are best understood in cortex where they surround fast-spiking inhibitory interneurons, the vast majority of studies examining PN structure and function focus on GABAergic cells (Härtig et al., 1992; Hensch, 2005; Nowicka et al., 2009; Karetko-Sysa et al., 2014; Testa et al., 2019; Rumschlag et al., 2021). In the brainstem, PNs are present in many species and commonly surround both GABAergic and non-GABAergic cells (Bertolotto et al., 1996; Hilbig et al., 2007; Foster et al., 2014; Fader et al., 2016; Beebe et al., 2018; Beebe and Schofield, 2018; Heusinger et al., 2019; Mansour et al., 2019; Sucha et al., 2019). We have previously demonstrated that PNs are upregulated with age in the IC on both GABAergic and non-GABAergic cells (Mafi et al., 2020). Furthermore, a proportion of the increasing IC PN population occurs on both GABAergic and non-GABAergic cells that project to the medial geniculate body (MG; Mafi et al., 2021). In the current study we demonstrate that the descending IC-CN pathway, which is glutamatergic, is also increasingly surrounded by PNs with age. Interestingly, the percentage of IC-CN cells and glutamatergic IC-MG cells that had a PN at young age (IC-CN- ~9.5%; IC-MG- ~12%) and old age (IC-CN- ~19%; IC-MG- ~18%) was remarkably similar (Mafi et al., 2021; current study). Thus, the upregulation of PNs in the aging IC is not unique to ascending pathways, but may rather be a property of IC cells more generally. Perhaps the upregulation on these subsets of IC cells is driven by shared properties of their inputs and/or their targets.

In the current study we found retrogradely labeled cells, with PNs, in both the ipsilateral and contralateral ICc and IClc. Across numerous species, there is an agreeance that the IC-CN pathway is bilateral with an ipsilateral dominance (cat-Hashikawa, 1983; guinea pig-Schofield, 2001; rat-Okoyama et al., 2006; mouse-Milinkeviciute et al., 2017). In the FBN rat we found a slight ipsilateral dominance (56.9%). When we examined the distribution across the subdivisions, most cells were in the ICc followed by the IClc, and then the ICd. A slight ipsilateral dominance was present in both the ICc-CN and the IClc-CN pathways. Although not presented in the Results as the few ICd-CN cells were not netted, this pathway also had an ipsilateral dominance (~61%). Broadly, our distribution of IC-CN cells agrees with a very detailed report in mouse (Milinkeviciute et al., 2017). In the current study the majority of IC-CN cells originated in the ICc, however another study in rat reported that the majority of IC-CN cells were found in the IClc layer III (Faye-Lund, 1986). The difference could be explained by a number of factors such as how the border between IClc layer III and the ICc was determined, the rostrocaudal extent of the IC that was analyzed, uptake properties of the tracers and strain of rat. In the context of the current study, we found netted IC-CN cells in the ipsilateral and contralateral ICc and IClc. Each IC location experienced an increase in PNs during old age on cells that project to the CN (Table 1). These data are similar to previous studies demonstrating that a subpopulation of lemniscal and non-lemniscal IC-MG cells have PNs (Beebe et al., 2018; Mafi et al., 2021). The potential functions of PNs on lemniscal and non-lemniscal IC-CN cells are discussed below.

To our knowledge the current study is the only one to demonstrate an age-related increase of PNs on a subcortical pathway that is largely, if not exclusively, glutamatergic. However, an age-related increase of PNs on excitatory populations of neurons is not unique. We demonstrated that excitatory IC-MG cells are increasingly surrounded by PNs during old age (Mafi et al., 2021). Additionally, an aging study of sensory cortex demonstrated PN increases specifically on non-GABAergic cells (Karetko-Sysa et al., 2014). One of the more notable characteristics of PNs is that they help support the ionic environment underlying the regulation of fast spiking in GABAergic cells (Corvetti and Rossi, 2005; Suttkus et al., 2012; Cabungcal et al., 2013; de Vivo et al., 2013; Blosa et al., 2015; Balmer, 2016; Sorg et al., 2016). Do PNs provide the same support for non-GABAergic populations? Regardless of aging, PNs that surround excitatory IC cells rather than inhibitory cells may differ in function. In the IC, larger GABAergic cells receive numerous vGlut2 synapses that form a precise ring around the soma (Ito et al., 2009). Perhaps PNs on larger GABAergic cells comprise CSPGs that form tighter nets in order to inhibit structural plasticity and “lock” the vGlut2 synapses in place while PNs on excitatory cells protect against excitotoxicity by regulating ionic concentrations and supporting synaptic plasticity.

Lastly, it is interesting to note that unlike our previous study on PNs surrounding the aging IC-MG pathway, the number of retrogradely labeled cells did not significantly differ between the young and old cases (Table 1; Mafi et al., 2021). Many criteria contribute to the effectiveness of a given retrograde tracer, and the ultimate population of labeled cells in a given nucleus can vary considerably from case to case (Schofield, 2008). However, in our two studies that use the same tracers in the same strain of rat at the same ages, perhaps retrograde transport is more effected by aging in the IC-MG pathway than the IC-CN pathway. Another possible interpretation is that the IC-MG pathway reduces in size with age while the IC-CN pathway does not. Further investigation of this question is needed.

### 4.3 Perineuronal nets and aging

The current study largely agrees with our previous reports on PNs in the aging IC in that a significant upregulation occurred in our old age group across multiple IC subdivisions (Mafi et al., 2020, 2021). These findings are also applicable to IC cells that project out of the IC as an increasing subset of the glutamatergic IC-CN and the glutamatergic IC-MG cells are netted at old age (Mafi et al., 2021, current study). Given that glutamatergic IC cells also project to a number of other nuclei (e.g., superior colliculus and superior olivary complex), it would be interesting to investigate whether all output pathways of the IC are undergoing similar changes to structural and/or synaptic plasticity during aging (see reviews, Thompson, 2005; Wenstrup, 2005). In order to understand the function of the PNs on these aging cell populations, additional studies examining the CSPG composition of the PNs will be needed. It is quite possible that PNs upregulated in old age on separate (e.g., glutamatergic vs. GABAergic; ascending vs. descending) neural populations in the IC comprise different CSPGs and/or have different functions. Interestingly, the lengths of excitatory synapses increase in the aging IC (Helfert et al., 1999; Mafi et al., 2022). Excitatory active zone lengthening during aging has been postulated as a compensatory mechanism (Adams, 1987; Levine et al., 1988). Perhaps the increase of specific CSPGs helps maintain these longer excitatory synapses during old age by preserving synapses providing critical excitatory feedback to the CN.

Studies have demonstrated that PNs are critically important to protect neurons against oxidative stress (Morawski et al., 2004; Suttkus et al., 2012, 2014; Cabungcal et al., 2013; Liguori et al., 2019). Redoxactive iron is scavenged and bound by PNs, thus the oxidative potential in the cell’s microenvironment is reduced (Suttkus et al., 2014). Specifically, aggrecan, the primary CSPG component of PNs, including PNs in the IC, has known neuroprotective effects against oxidative stress (Suttkus et al., 2014; Beebe and Schofield, 2018). IC a number of other proteoglycans have been shown to support synaptic function. Aggrecan has been shown to stabilize synaptic structure, while brevican and neurocan are important for the maintenance of inhibitory and excitatory synapses (Zaremba et al., 1989; Gottschling et al., 2019; Schmidt et al., 2020). The proteoglycan neurocan is also known for its roles in the development of PNs, while phosphacan is better understood in the aging brain (Nishiwaki et al., 1998; Gottschling et al., 2019; Schmidt et al., 2020). Understanding the molecular composition of PNs on IC-CN cells may reveal whether the upregulated PNs are subserving different functional roles throughout aging.

PNs are perhaps best known for their role in reduced structural plasticity at young and old ages (Corvetti and Rossi, 2005; Frischknecht et al., 2009; Karetko-Sysa et al., 2014; Sonntag et al., 2015; see review: Duncan et al., 2019). Compromising PNs can lead to increased plasticity in visual, fear conditioning, and learning and memory circuits (Pizzorusso et al., 2006; see reviews: Wang and Fawcett, 2012; Duncan et al., 2019; Testa et al., 2019). Studies of subcortical nuclei, hippocampus and non-auditory cortex agree with the current study in that the quantity of PNs increase with age (Tanaka and Mizoguchi, 2009; Yamada and Jinno, 2013; Fader et al., 2016; Ueno et al., 2018; Quraishe et al., 2019; Mafi et al., 2020). Yet, activated microglia facilitate plaque-dependent PN loss associated with Alzheimer’s Disease (Crapser et al., 2020). This paradox (increased inflammation coupled with region-specific increase in PNs with aging) has yet to be reconciled. Studies looking at the effects of aging on MMP-9 (an enzyme responsible for PN degradation) expression and/or microglial activation in the aging inferior colliculus could help resolve this discrepancy. The lengths of excitatory synapses increase in the aging IC (Helfert et al., 1999; Mafi et al., 2022). Excitatory active zone lengthening during aging has been postulated as a compensatory mechanism (Adams, 1987; Levine et al., 1988). Perhaps the increase of PNs on IC cells is helping maintain these longer excitatory synapses during old age by preserving synapses providing critical excitatory feedback to the CN. Either way, we conclude that robust changes to synaptic and/or structural plasticity are taking place on lemniscal and non-lemniscal excitatory circuits reaching the CN from the aged IC.

### 4.4 Potential functional roles of PNs in age-related hearing loss

Deficits in temporal processing in the auditory system are commonly viewed as a key contributor to age-related hearing loss (see reviews Frisina, 2009; Liberman, 2017; Syka, 2020). Specifically, temporal precision of IC cells is diminished when GABAergic neurotransmission is interrupted (Palombi and Caspary, 1996a,b; Frisina, 2001; LeBeau et al., 2001; Walton et al., 2002; Parthasarathy and Bartlett, 2011; Caspary and Llano, 2018; Parthasarathy et al., 2018; Syka, 2020). Perhaps the most direct manner in which GABAergic neurotransmission is interrupted in the aging IC is the loss of GABAergic synapses (Helfert et al., 1999; Caspary et al., 2008; Syka, 2020). However, as GABAergic synapses are lost with age in the ICc and IClc, the proportion of available GABAergic synapses targeting larger dendrites increases during middle age (Helfert et al., 1999; Mafi et al., 2022). Given that this reorganization of GABAergic synapses is occurring during an age when age-related hearing deficits are rare in the rat models of aging, it has been hypothesized that the reorganization is a compensatory mechanism that supports continued “normal” hearing with aging (Helfert et al., 1999; Caspary et al., 2008). ICc and IClc cells also alter the subunit composition of postsynaptic GABA_A_ receptors and maximally express specific subunits during middle age (e.g., GABA_A_ α_2_ and γ_1_ subunits; Milbrandt et al., 1996, 1997; Caspary et al., 1999; Kelly and Caspary, 2005; Robinson et al., 2019). The increase of the GABA_A_ α_2_ and γ_1_ subunits can allow a given GABAergic cell to become more sensitive to GABA and may occur in response to diminished excitation arriving from the periphery (Milbrandt et al., 1997; Caspary et al., 1999, 2008). PNs subserve many functions (e.g., stabilizing high-rate synaptic transmission, forming critical periods, and regulating oxidative stress) of synaptic plasticity (Bukalo et al., 2001; Balmer et al., 2009; de Vivo et al., 2013; Blosa et al., 2015; Sonntag et al., 2015; Bosiacki et al., 2019; Testa et al., 2019). The increasing population of PNs may be a homeostatic mechanism necessary to stabilize, “lock,” and maintain synaptic integrity as pre- and postsynaptic GABAergic changes occur throughout the aging IC. Thus, PNs forming during aging may serve to stabilize existing circuits by maintaining the correct excitatory-inhibitory balance throughout adulthood; indeed, it is not until old age that significant deficits to temporal and speech processing occur (Pichora-Fuller and Souza, 2003; Walton, 2010; Ruggles et al., 2011; Karetko-Sysa et al., 2014; Füllgrabe et al., 2015). Further studies will be necessary to determine if IC-CN cells undergo the aforementioned pre- and postsynaptic GABAergic changes.

Our knowledge of PN functions in the subcortical auditory nuclei, let alone in the context of aging, is extremely limited (Bertolotto et al., 1996; Hilbig et al., 2007; Myers et al., 2012; Foster et al., 2014; Sonntag et al., 2015; Beebe et al., 2016, 2018; Fader et al., 2016; Beebe and Schofield, 2018; Heusinger et al., 2019; Mansour et al., 2019; Quraishe et al., 2019; Sucha et al., 2019; Mafi et al., 2020). It has been shown that neonatal conductive hearing loss can degrade PN development in the superior olive (Myers et al., 2012). Also, a study in the auditory thalamus determined that a cell’s temporal acuity and synchronization to faster repetition rates improved during aging when surrounded by a PN (Quraishe et al., 2019). Specifically, temporal processing was improved in the ventral (lemniscal) and medial (non-lemniscal) auditory thalamus (Quraishe et al., 2019). It appears that in the aging auditory thalamus, PNs serve a role related to their ability respond precisely to temporally complex stimuli (Cabungcal et al., 2013; Blosa et al., 2015; Balmer, 2016). However, as mentioned above, temporal acuity deteriorates with age in the IC despite our finding of increased PNs (see reviews: Caspary et al., 2008; Caspary and Llano, 2018; Syka, 2020). It is possible that the increased PN populations supports different functional roles (i.e., oxidative stress reduction or synaptic stabilization) in different subcortical auditory nuclei. Differences in species may also explain some functional differences in PNs. For example, the current study uses a model (FBN rat) that undergoes age-related changes to its peripheral auditory system (Cai et al., 2018), while the aged mice in Quraishe et al. (2019) did not have substantial changes in their peripheral auditory system.

As PNs decrease structural plasticity, it is possible that IC cells undergoing compensatory synaptic changes in middle age are structurally inhibited in old age if they have a PN. This may lead to an imbalance of inhibition and excitation that underlies characteristics of age-related hearing loss (Palombi and Caspary, 1996a,b; Walton et al., 1998, 2002; Frisina, 2001; Parthasarathy and Bartlett, 2011). As lemniscal and non-lemniscal IC nuclei send excitatory inputs to both dorsal cochlear nuclei, the increase of PNs on IC-CN cells may have very broad effects (Andersen et al., 1980; Conlee and Kane, 1982; Shore et al., 1991; Caicedo and Herbert, 1993; Malmierca et al., 1996; Saint Marie, 1996; Schofield, 2001; Milinkeviciute et al., 2017). In agreeance with Schofield (2001) and Milinkeviciute et al. (2017), we find inputs to the CN from each subdivision of the IC, with the largest source being the ICc. For reasons that are unclear, cells from the dorsal cortex of the IC that project to the CN were never netted at any age. Similar to Faye-Lund (1986), we find that our IClc-CN cells largely reside in layer III of IClc. Thus, most of our IC-CN cells are in regions receiving lateral lemniscal input (Oliver, 2005). It appears that PNs are likely altering the plastic environment of IC cells that receive lemniscal input and then provide excitatory feedback to the earliest synapses in the central auditory pathway.

As this is the first study to demonstrate PNs on a subcortical descending auditory (or possibly any sensory modality) pathway, the specific inputs and outputs of IC-CN cells need to be better understood to unravel how PNs may affect hearing. Fortunately, a recent study by Balmer and Trussell (2022) greatly detailed how the IC targets all but one cell type in the DCN. Thus, the IC-CN projection is setup to potentially affect excitation and inhibition in the DCN via a number of circuits that may contribute to improved frequency detection, enhancing bottom-up sensitivity to auditory nerve input, frequency filtering and central gain (Balmer and Trussell, 2022). As the DCN is often a nucleus of interest in the maintenance of tinnitus, the upregulation of PNs on IC-CN cells at old age may lead to an imbalance of excitation and inhibition that could underlie elements of hyperexcitability in the CN (Kaltenbach and Godfrey, 2019; Balmer and Trussell, 2022). Determining whether the increasing PN populations surround IC cells that target specific DCN cell types will provide more clarity regarding the possible functions underlying plastic changes later in life. While the cellular targets of IC-CN projections are well-characterized, the inputs to IC-CN cells are less clear. Given the robust commissural projection between the left and right, it would not be surprising if an IC-CN cell conveys information from both ICs (Saldaña and Merchán, 1992). The IC-CN projection also targets the CN bilaterally, so it is conceivable that the final inputs to the either CN from either IC may represent processed signals that originated in the contralateral, ipsilateral or both ears. Lastly, IC cells that have descending projections to the CN are modulated by descending input from the auditory cortex (Schofield and Coomes, 2006). It appears likely that PNs are affecting the synaptic and structural plasticity of cortical, commissural, and local inputs onto aging IC-CN cells.

The current study identifies a second output pathway of the IC that is increasingly surrounded by PNs at old age (Mafi et al., 2021). As only a subset (~20%) of each pathway has a PN at old age, perhaps there is a shared functional role(s) these netted cells serve in their respective pathways. Furthermore, it is of interest to determine whether upregulated nets share the same molecular composition as PNs in younger animals and across circuits. Future studies are required to determine if the PNs at old age are having a beneficial or detrimental effect on the balance of inhibition and excitation. Ultimately, PNs at any age must have a broad impact on hearing as both descending and ascending pathways from the IC undergo age-related changes in their plasticity.

## Data availability statement

The original contributions presented in the study are included in the article/supplementary material, further inquiries can be directed to the corresponding author.

## Ethics statement

The animal study was approved by the Northeast Ohio Medical University IACUC. The study was conducted in accordance with the local legislation and institutional requirements.

## Author contributions

LA: Data curation, Investigation, Writing – original draft, Writing – review & editing. AO: Data curation, Investigation, Writing – review & editing. MI: Investigation, Writing – review & editing. AW: Investigation, Writing – review & editing. NT: Conceptualization, Methodology, Supervision, Writing – review & editing. AM: Conceptualization, Data curation, Formal analysis, Methodology, Writing – review & editing. NB: Writing – original draft, Writing – review & editing. JY: Data curation, Formal analysis, Software, Writing – original draft, Writing – review & editing. JM: Conceptualization, Data curation, Funding acquisition, Investigation, Methodology, Project administration, Resources, Supervision, Validation, Writing – original draft, Writing – review & editing.

## Glossary

CN: cochlear nucleus
CSPG: chondroitin sulfate proteoglycans
DCN: dorsal cochlear nucleus
ECM: extracellular matrix
FBN: Fischer Brown Norway
GABA: gamma-aminobutyric acid
GAD: glutamic acid decarboxylase
IC: inferior colliculus
ICc: central nucleus of the inferior colliculus
ICd: dorsal cortex of the inferior colliculus
IClc: lateral cortex of the inferior colliculus
MG: medial geniculate body
NT: NeuroTrace
PNs: perineuronal nets
VCN: ventral cochlear nucleus
WFA: *Wisteria floribunda* agglutinin
